# The Meharry Medical College

**Published:** 1897-03

**Authors:** 


THE MEHARRY MEDICAL COLLEGE, NASHVILLE, TENN, held its 21st annual commencement February 1st, ult., graduating thirty-four students out of a class of one hundred and fifty-one. The valedictory was delivered by David Wilmot Dunn, A. B., of Mississippi. His thesis was in Latin, and was entitled "*Scientia Medicinæ: Retrospectus et Predictum.* The valedictory to the class in pharmacy was delivered by Miss Belina A. Moore, of Alabama, the only woman in the class.

The Meharry Medical College, which embraces the branches of pharmacy and dentistry, is the largest college of the kind in the world for the education of negroes. It is doing a good work. Four courses, in four separate years, are required, as a condition to graduation. The institution was established in 1876, by Rev. Samuel Meharry, of Indiana, of Irish descent, who is still living, at the age of 85, in the town of Lafayette, Indiana.

